# Rhizosphere microbiome dynamics and hormonal interactions regulating tiller development in sugarcane cultivars

**DOI:** 10.1038/s41598-026-38474-y

**Published:** 2026-03-22

**Authors:** Qinyu Lu, Shimiao Chen, Bin Shan, Ailin Wei, Yuhuan Luo, Lanfang Wu, Qiang Jiang, ZhenDong Chen

**Affiliations:** 1 Guangxi Baise Modern Agriculture Technology Research and Extension Center, Management Committee of Baise National Agricultural science and Technology Zone of Guangxi, Baise, 533612 China; 2 Guangxi Academy of Agricultural Science, Nanning, 530001 China; 3 Guangxi Subtropical Crops Research Institute, Nanning, 530001 China; 4 Baise Institute of Agricultural Sciences, Baise, 533612 China; 5 Guangxi Zhuang Autonomous Region, Seed Station of the Bureau of Agriculture and Rural Affairs, Tianyang District, Baise City, Baise, 533612 China

**Keywords:** Sugarcane, Rhizosphere microbiome, Hormonal regulation, Tiller development, Plant–microbe interactions., Microbiology, Plant sciences

## Abstract

Sugarcane tillering is a key determinant of crop productivity, yet the integrated roles of rhizosphere microbiome dynamics, nutrient status, and hormone signaling in regulating tiller development remain poorly understood. Here, we compared rhizosphere microbial communities, endogenous hormone profiles, and nutrient element concentrations in sugarcane cultivars with contrasting tillering capacities. High-tillering varieties exhibited significantly greater microbial diversity and more complex co-occurrence network structures in the rhizosphere, characterized by enrichment of Acidobacteriota, Chloroflexi, and Planctomycetes and functional pathways related to nitrogen fixation, phosphorus solubilization, and auxin biosynthesis. In contrast, low-tillering varieties harbored simplified, stress-adapted microbial consortia and prioritized pathways linked to oxidative stress response and heavy metal detoxification. Hormonal analysis revealed that high-tillering cultivars maintained higher levels of growth-promoting hormones—particularly auxin (IAA) and active cytokinins—in tiller buds while low-tillering cultivars accumulated elevated abscisic acid (ABA) and inactive cytokinin conjugates. Nutrient analysis indicated that high-tillering genotypes possessed higher nitrogen and phosphorus contents, supporting vigorous axillary bud activation and shoot proliferation, whereas low-tillering varieties accumulated more zinc and manganese, potentially reflecting stress adaptation. Network-level integration of microbial, hormonal, and nutrient profiles underscored genotype-specific feedback between rhizosphere microbiota and plant physiological states, highlighting modular associations that link microbial hubs with tissue-specific nutrient and hormone signatures. Our findings reveal a systems-level mechanism by which rhizosphere microbial community structure and function interact with plant-nutrient–hormonal status to regulate tillering in sugarcane. These insights provide a basis for microbiome-informed strategies to enhance sugarcane productivity through integrated nutrient–hormonal–microbe management.

**Supplementary Information:**

The online version contains supplementary material available at 10.1038/s41598-026-38474-y.

## Introduction

Globally, sugarcane accounts for approximately 75% of the world’s sugar production, making it the primary source of sugar^1^. Beyond its traditional use in sugar production, sugarcane is a key feedstock for biofuel generation—such as ethanol and bio-butanol—and industrial enzymes, paper, and bioplastics^2^. In China, sugarcane plays a significant role in the agricultural economy, contributing about 85–90% of the nation’s total sugar output. In certain provinces, the sugar industry is a vital economic pillar; for example, it accounts for 10–11% of Guangxi’s GDP^3^.

Despite its status as a pillar industry, sugarcane production is undergoing significant changes. In recent years, the total area dedicated to sugarcane cultivation has declined due to competition from alternative crops and constraints on arable land, reducing total production from 115.75 million tons in 2012 to 103.38 million tons in 2022. While output in Guangxi has remained stable, regions such as Yunnan and Guangdong have experienced notable declines^3^. Moreover, declining cultivation areas, extreme weather events (e.g., typhoons), and competition from other crops continue challenging the industry^4^. Additionally, high production costs—stemming from low mechanization rates and rising input prices—adversely affect farmers’ profitability and discourage the expansion of cultivation areas^3^. Despite these challenges, sugarcane remains a crucial crop for China’s agricultural economy, and efforts to enhance production and sustainability may further strengthen the industry’s prospects.

Sugarcane tillering directly and significantly influences yield by determining the number of stalks available for harvest, a key factor in overall productivity. Tillering refers to producing shoots (tillers) from the plant’s base, which subsequently develop into millable stalks—the primary contributors to sugarcane yield. Consequently, more tillers typically result in a more significant stalk population at harvest, directly enhancing the crop’s yield potential^5^. Moreover, tillers that survive competition and environmental stress develop into mature stalks that contribute to the final harvest. Drought-tolerant varieties often maintain higher tiller numbers under stress, contributing positively to yield even under suboptimal conditions^6^. Tillering is closely linked to root development, as robust root systems support better nutrient uptake and water absorption, sustaining healthy tiller growth and increasing above-ground dry matter accumulation^7^.

However, excessive or poorly managed tillering can lead to inefficient resource use without proportional yield gains^8^. Over-tillering may divert soil nutrients—especially under nutrient-limited conditions—resulting in insufficient nutrient allocation to each stalk and ultimately compromising yield and quality^8^. Additionally, dense tillering can form a closed canopy, limiting light penetration to lower leaves and reducing overall photosynthetic efficiency. In contrast, an optimal tiller density improves canopy structure, enhances light utilization, and increases biomass and sugar yield^9^. Excessive tillering also elevates water demand, severely impacting sugarcane growth and yield in arid regions^10^. Recent studies have demonstrated that controlling tillering in sugarcane is crucial for optimizing yield and overall crop performance.

In regulating tillering signals in sugarcane, hormones play a crucial role throughout the process. Strigolactones (SLs) act as central inhibitors by suppressing axillary bud outgrowth; elevated SL levels in Saccharum spontaneum reduce tillering, whereas lower SL levels in *S. officinarum* correlate with higher tiller production^11^. Additionally, auxins and cytokinins maintain a balance in tiller growth: auxins (e.g., IAA) produced in the shoot apex inhibit bud activation, while cytokinins promote bud outgrowth, counteracting auxin’s inhibitory effects^12^. Furthermore, abscisic acid (ABA) accumulates in senescing tillers—accelerating cell membrane degradation and lipid peroxidation—whereas live tillers exhibit undetectable ABA levels, highlighting its role in tiller senescence^13^.

Nutrient availability is also critical for sugarcane growth, particularly during the tillering stage, which is essential for establishing a dense crop stand. Nitrogen supply significantly affects a crop’s tillering ability. Studies have shown nitrogen can influence rice tillering by regulating sugar transporter proteins (e.g., *OsSTP28*)^14^. Nitrogen supply suppresses the expression of *OsSTP28*, leading to extracellular glucose accumulation in the tiller buds inhibiting tillering. Phosphorus promotes root development, early shoot growth, and tillering, enhancing internode elongation and crop establishment. Potassium is crucial for biomass production and sugar accumulation by facilitating the transport of sugars from leaves to stalks, thereby increasing sucrose content in mature stalks^6^. Magnesium, a central component of chlorophyll molecules, enhances photosynthesis, while sulfur improves soil health, boosts chlorophyll content, and contributes to protein synthesis^15^. Calcium strengthens plant structure, supporting root and stalk development^16^, and boron is essential for carbohydrate metabolism and nucleic acid synthesis during early growth^17^. Finally, zinc promotes strong rooting and shoot development^18^.

From the discussion above, it is evident that rhizospheric microorganisms play a significant role. For example, certain rhizosphere bacteria—such as *Azotobacter* and *Azospirillum*—can fix atmospheric nitrogen and convert it into plant-available forms, thereby reducing reliance on nitrogen fertilizers^19,20^. Nitrogen is also a key precursor for synthesizing important hormones like auxins and cytokinins, indirectly influencing hormone levels. Bacteria such as *Pseudomonas chlororaphis*, *P. fluorescens*, and *Bacillus cereus* can solubilize insoluble phosphorus in the soil, increasing its availability and promoting plant growth^21^. Phosphorus is critical for plant energy metabolism and signal transduction, affecting the synthesis and response of hormones. Moreover, some microorganisms can directly produce or modulate plant hormones—such as auxin (IAA), cytokinins, and gibberellins—thereby influencing plant growth and development^22^. For instance, *Gluconacetobacter diazotrophicus* has been shown to affect sugarcane’s defense response against the pathogen *Xanthomonas albilineans*^23^.

Additionally, the performance of rhizospheric microorganisms varies among different sugarcane cultivars, reflecting distinct microbial interactions that underpin their resilience and nutrient dynamics. For instance, drought-tolerant cultivars like ZZ9 maintain stable bacterial diversity under water deficit by preemptively hosting drought-resistant taxa (e.g., Actinobacteria and Alphaproteobacteria) and establishing complex Bacilli‐dominated networks, whereas drought‐sensitive cultivars such as GT39 exhibit reduced diversity and primarily recruit drought‐resistant microbes like Sphingomonadales during stress, with weaker links between soil enzymes and microbial activity^24^. Furthermore, root exudate composition plays a crucial role in shaping the rhizosphere: cultivars like ZZ9 secrete exudates that enrich nitrogen‐fixing Rhizobiales and phosphate‐solubilizing *Streptomycetales*, thereby optimizing nutrient cycling, while sensitive cultivars show diminished exudate-mediated enrichment, potentially leading to nutrient deficiencies^25^. Genetically modified cultivars overexpressing drought-resistance genes (e.g., DREB) further boost exudate production, enriching Actinobacteria and improving nitrogen fixation in intercropped soybean^26^. Moreover, intercropping significantly shifts the microbial community; for example, ZZ9 intercropped with soybean develops a bacterial profile more akin to that of soybean roots^25^, with increased Flavobacteria and Betaproteobacteria, while GM sugarcane intercropped with soybean enhances root interactions by growing populations of *Taibaiella* and *Rhodanobacter*, which are associated with organic matter decomposition^26^. Finally, temporal dynamics across growth stages—modulated by factors such as pH and total soil nitrogen—drive shifts in microbial composition, with tolerant cultivars stabilizing populations of Acidobacteria and Chloroflexi during the elongation stages to facilitate efficient carbon cycling^27^.

In this study, we evaluate the rhizospheric microorganism populations and rhizosphere characteristics in sugarcane varieties with differing tillering capacities and analyze the associated plant hormone responses. We aim to elucidate the relationship between microbial communities and tillering and to assess how microbial recruitment capacity influences tillering ability. Ultimately, this research seeks to provide valuable insights into fertility management in sugarcane production.

## Material and method

Using the Iijima et al. method^28^, soil samples were collected from sugarcane cultivars—two high-tillering and two low-tillering—from the same nursery garden during the peak tillering period. A portion of each soil sample was quickly frozen in liquid nitrogen and stored at − 80 °C for microbiome analysis. Additionally, shoots from the corresponding plants were collected; some were flash-frozen in liquid nitrogen and stored at − 80 °C for endogenous hormone analysis, while the remaining samples were dried and ground for the analysis of nutritive element content.

### Plant materials

Sugarcane cultivars with contrasting tillering capacity were used in this study. High-tillering and low-tillering genotypes were obtained from the Guangxi Academy of Agricultural Sciences (Nanning, Guangxi, China). Plants were maintained and propagated under standard greenhouse conditions prior to experimental treatments. For each sugarcane cultivar, three independent plants were sampled as biological replicates (*n* = 3). From each plant, rhizosphere soil was collected from three representative root segments and pooled into one composite sample per plant. All subsequent analyses (microbiome sequencing, nutrient element determination, and hormone quantification) were conducted using these three biological replicates.

### Microbiome analysis

Soil microbial community structure was analyzed using the method of Xi et al.^29^. Total DNA was extracted from soil samples using the Soil Genomic DNA Extraction Kit (Solarbio, China), following the manufacturer’s protocol. For bacterial community analysis, the V3–V4 hypervariable region of the 16 S rRNA gene was amplified using the primer pair 341 F/805R. PCR amplicons were purified, quantified, and pooled at equimolar concentrations before paired-end sequencing on an Illumina MiSeq platform, outsourced to Sangon Biotech (China). Raw sequence data were processed using QIIME2 with the DADA2 pipeline for quality filtering, denoising, and chimera removal, followed by taxonomic assignment using the Greengenes database. Microbial diversity was assessed by principal coordinate analysis (PCoA) to visualize community differences among samples. Collinearity analysis was performed using the R package circlize, while functional predictive analysis was conducted with Tax4Fun2 and further analyzed using PCA. Additional plots, including heatmaps and Venn diagrams, were generated using various R packages (ggraph, VennDiagram, corrplot, etc.).

### Endogenous hormone content analysis

Endogenous hormone contents were determined following the method of Kasote et al.^30^ with minor modifications. A 100 mg sample of frozen plant tissue was immediately ground into a fine powder in liquid nitrogen. The powdered tissue was then transferred to a 2 mL microcentrifuge tube and extracted with 9.8 mL of a 99:1 (v/v) methanol–acetic acid mixture. After vigorous vortexing and centrifugation at 13,000×g for 5 min at 4 °C, the supernatant was filtered through a 0.22 μm nylon syringe filter. A 2 µL aliquot of the filtered extract was subsequently injected into a UHPLC-MS/MS system (TSQ Quantis, Thermo Scientific, USA) equipped with a C18 column. The analytes were separated using a binary gradient of 0.1% formic acid in water and acetonitrile (mobile phase conditions are detailed in Table S1). At the same time, mass spectrometric detection was performed in multiple reaction monitoring (MRM) mode (ion pairs are listed in Table S2) using electrospray ionization in both positive and negative modes.

### Analysis of nutrient elements within plant content

Nutrient element analysis in plant tissues was conducted using ICP-OES^31^. Dried plant samples were finely ground, and approximately 0.5 g of each sample was digested with a mixture of concentrated nitric acid and hydrogen peroxide in a microwave digestion system under controlled conditions. The resulting digest was diluted with deionized water and filtered through a 0.45 μm membrane filter to remove residual particulates. Essential macro- (e.g., N, P, K, Ca, Mg) and microelements (e.g., Fe, Zn, Mn, Cu) were subsequently quantified using ICP-OES (EXPEC 6500, EXPEC Technology, China). Calibration curves were constructed using standard solutions, and the accuracy of the measurements was validated using certified reference materials, ensuring reliable assessments of nutrient element concentrations in the plant tissues.

### Microbial co-occurrence networks analysis

Microbial co-occurrence networks integrating environmental variables were constructed using the ggClusterNet package in R, following the approach of Qiu et al.^32^. High-throughput sequencing data were quality filtered and processed to generate an OTU/ESV table. In contrast, environmental variables were normalized and compiled from corresponding metadata. Pairwise Spearman correlation coefficients between microbial taxa and ecological parameters were calculated, and only associations with *r* > 0.8 and *P* < 0.05 were retained to build the network. Global topological features—including network density, modularity, and average path length—were computed using the net_properties. Two functions and modules were delineated via the fast greedy clustering algorithm. Finally, the network was visualized using interactive tools such as Gephi, facilitating the interpretation of how environmental gradients influence microbial community structure and interactions.

### Compliance with ethical standards

All experimental research and field studies on sugarcane complied with institutional, national, and international guidelines and legislation. Permission for plant material collection and use was obtained from the Guangxi Academy of Agricultural Sciences.

## Result

### Analysis of rhizosphere microbial community structures

**Fig. 1:**
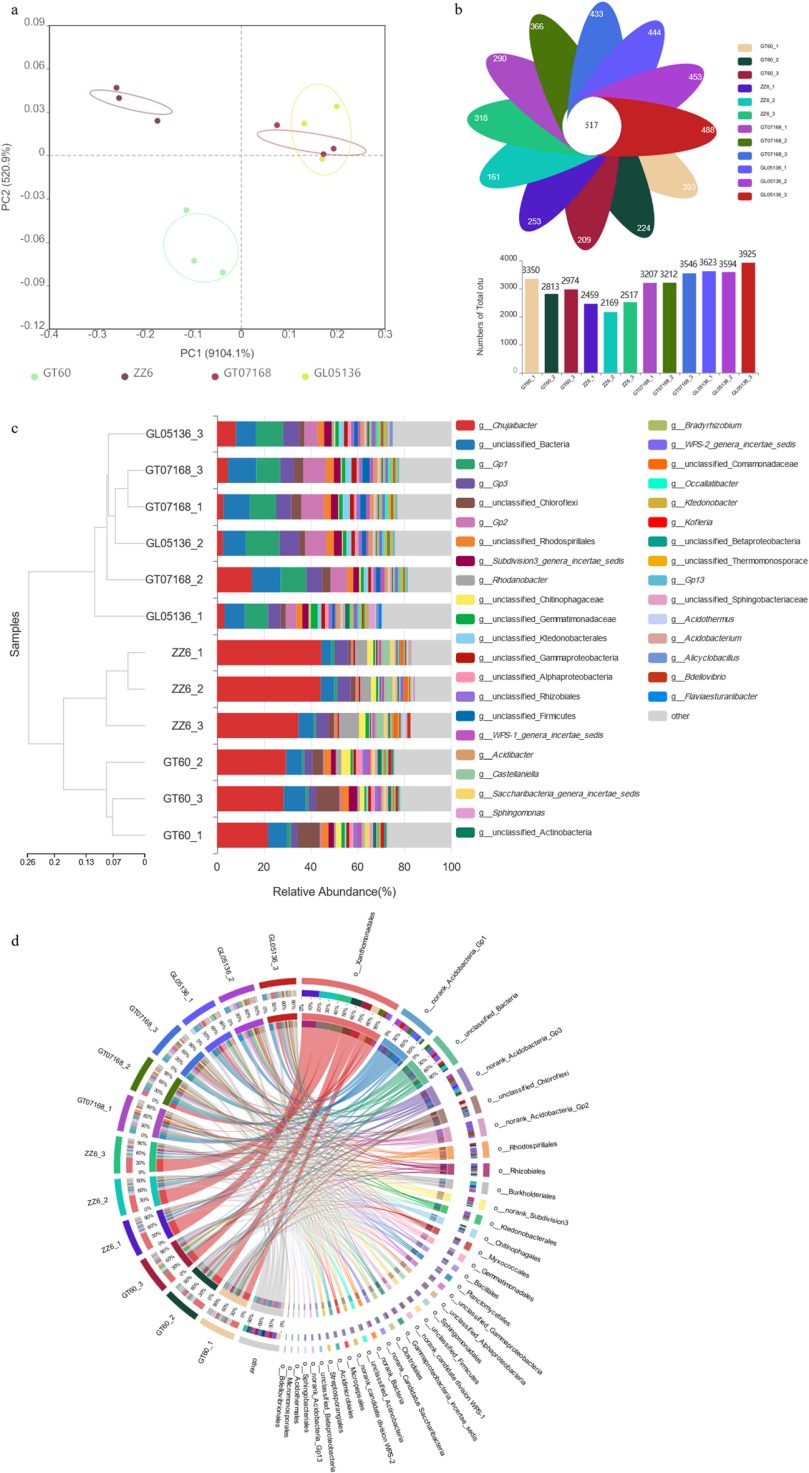
Comparative analysis of rhizosphere microbial communities in sugarcane cultivars with contrasting tillering capacities. (**a**) Principal component analysis (PCA) reveals distinct clustering of high- and low-tillering varieties based on rhizosphere microbiome composition. (**b**) Venn diagram displays core and unique OTUs among cultivars, highlighting greater microbial diversity in high-tillering genotypes. (**c**) Taxonomic composition at the phylum level shows shifts in dominant microbial groups between varieties. (**d**) Genus-level chord diagram illustrates more complex microbial associations in high-tillering cultivars.

The PCA results highlighted pronounced differences in rhizosphere microbial community structures between sugarcane varieties with contrasting tillering capacities (Fig. 1a). High-tillering varieties (GT07168 and GL05136) clustered tightly on the right side of the PCA plot, suggesting a highly similar microbial composition. In contrast, low-tillering varieties (GT60 and ZZ6) were separated from the high-tillering group and each other along PC1, indicating greater variability in their rhizosphere microbial profiles. Biological replicates showed relatively consistent distributions within each variety, reflecting stable community structures. However, GT60 samples displayed slightly more dispersion than other varieties, suggesting higher intra-varietal variability. These results demonstrate that high-tillering varieties maintain more conserved and distinct rhizosphere microbiomes, whereas low-tillering varieties exhibit greater divergence between and within genotypes.

Venn diagram analysis revealed a substantial core microbiome and distinct microbial subsets associated with different sugarcane varieties(Fig. 1b). A total of 518 operational taxonomic units (OTUs) were shared among all groups, representing the conserved core rhizosphere microbiome across cultivars with contrasting tillering capacities. Beyond the core, each group harbored unique microbial populations, with the number of unique OTUs ranging from 172 to 562. Notably, high-tillering varieties (GT07168 and GL05136) exhibited a markedly higher number of unique OTUs (up to 562) compared to low-tillering varieties (GT60 and ZZ6, with as few as 172 unique OTUs). This suggests that high-tillering cultivars maintain a richer and more diverse rhizosphere microbiome, potentially enhancing nutrient acquisition, promoting plant growth, and supporting increased tiller formation. Thus, while a conserved microbial core exists across sugarcane genotypes, variety-specific expansions in microbial diversity—particularly in high-tillering cultivars—may play a pivotal role in modulating tillering performance.

Analysis of the taxonomic composition revealed distinct rhizosphere community structures between high- and low-tillering sugarcane varieties (Fig. 1c). Proteobacteria dominated all samples, but its relative abundance was significantly higher in low-tillering cultivars (GT60 and ZZ6). In contrast, high-tillering varieties (GT07168 and GL05136) showed increased proportions of Acidobacteriota, Chloroflexi, and Planctomycetota—phyla often linked to nutrient cycling and plant growth promotion. Hierarchical clustering based on Bray–Curtis dissimilarity grouped samples primarily according to tillering capacity, underscoring a compositional shift aligned with plant performance.

At the genus level (Fig. 1d), chord diagram analysis indicated that high-tillering varieties harbored greater microbial diversity and more complex inter-taxon connections. Genera such as *Acidobacterium* (Acidobacteriota), *Bradyrhizobium*, and *Streptomyces* were more prevalent in high-tillering samples (Table S2). These taxa are known for their roles in organic matter degradation, nitrogen fixation, and phytohormone production, supporting rhizosphere nutrient dynamics and axillary bud development.

Conversely, low-tillering cultivars exhibited a more simplified microbial network dominated by stress-associated genera like *Rhodanobacter* and *Rhodospirillales*, which are often linked to heavy metal resistance, oxidative stress tolerance, and antibiotic production. This suggests that the microbial environment in low-tillering plants may be more oriented toward stress survival than active nutrient provisioning.

Although most taxonomic differences at the genus level did not reach statistical significance after multiple testing corrections, these observed trends align with known functional traits. Inferred microbial functions further support this divergence: high-tillering rhizospheres were enriched in pathways related to nitrogen metabolism, phosphorus solubilization, and auxin biosynthesis, while low-tillering rhizospheres were more aligned with stress adaptation, potentially limiting resource allocation for tiller formation.

### Differential functional profiling of rhizosphere microbiomes

**Fig. 2:**
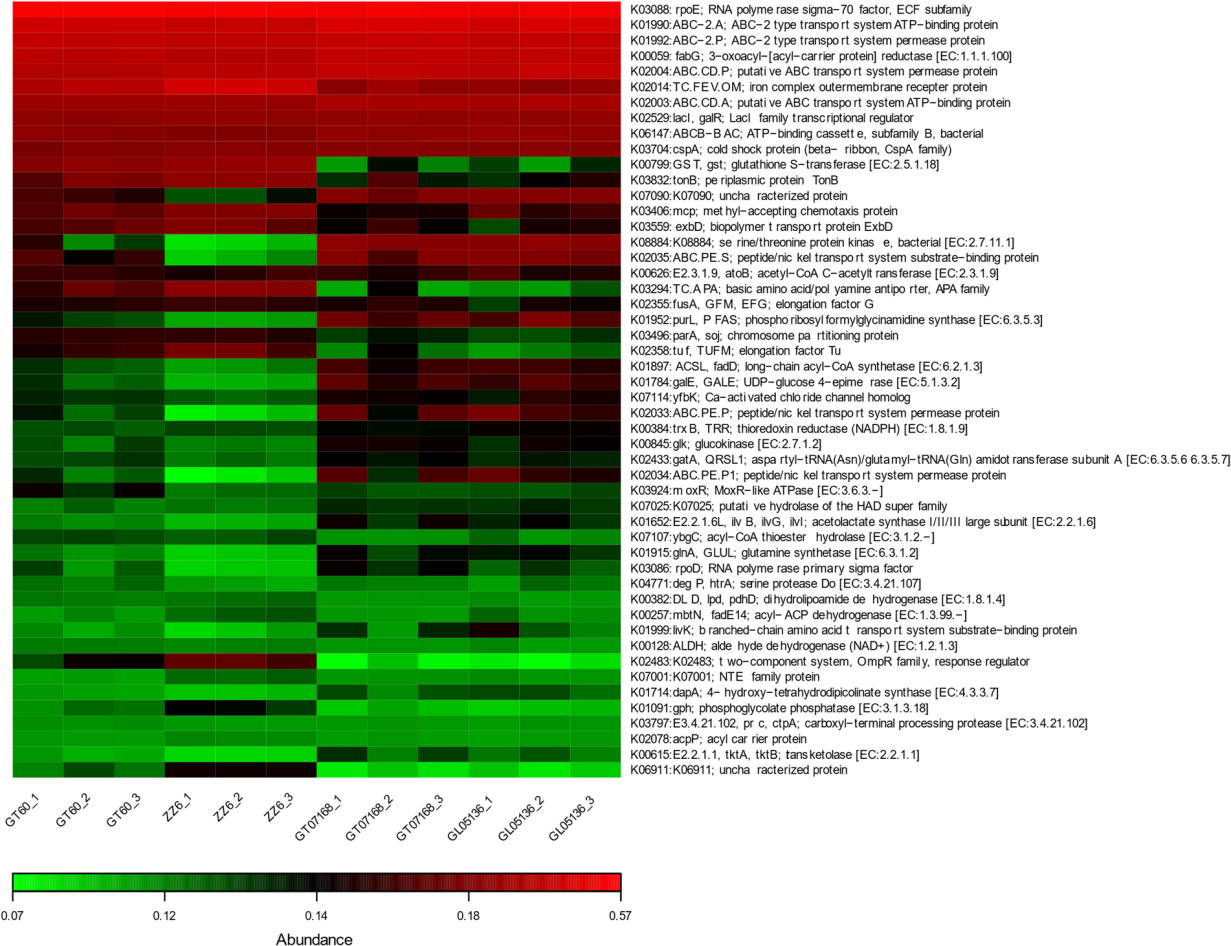
Functional profiling of rhizosphere microbiomes in sugarcane cultivars with high and low tillering capacity.

**Fig. 3:**
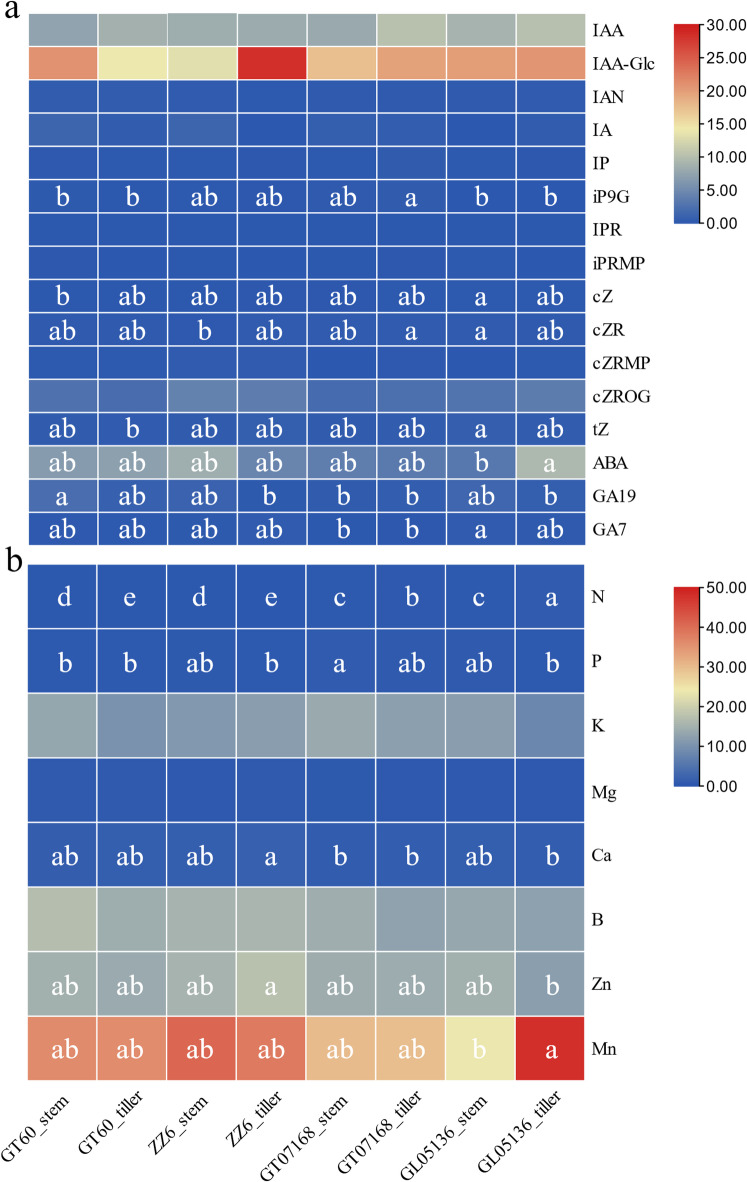
The heatmap of Endogenous hormone profiles (**a**) and Nutrient element (**b**) concentrations in different sugarcane cultivars and tissues. Heatmap colors represent the relative mean concentrations of endogenous hormones across cultivars and tissues. Lowercase letters are shown only for variables with statistically significant differences according to Duncan’s multiple-range test (*p* < 0.05), where “a” denotes the highest mean value and subsequent letters indicate decreasing levels. Cells without significant differences are left unlabelled.

The functional heatmap inferred from amplicon data (Fig. 2) offers a putative assessment of pathway differences across cultivars. Among these predicted functions, the top 50 most divergent pathways were mainly concentrated in the midsection, showing clear contrasts between high- and low-tillering varieties. Specifically, pathways related to nitrogen fixation, phosphate solubilization, carbohydrate metabolism (e.g., glycolysis, cellulose degradation), and auxin biosynthesis were positively associated with high-tillering cultivars (GT07168 and GL05136), but negatively related to low-tillering cultivars (GT60 and ZZ6). These functions are critically linked to nutrient acquisition and plant growth promotion, suggesting that microbiome-driven enhancement of nutrient availability and hormonal stimulation may underlie the vigorous tillering performance observed in high-tillering genotypes. Conversely, pathways associated with oxidative stress response, antibiotic resistance, and heavy metal detoxification exhibited positive associations with low-tillering varieties. This pattern indicates that the rhizosphere microbiomes of low-tillering cultivars may prioritize stress adaptation mechanisms over growth-supporting functions, potentially limiting their tillering capacity. These functional differences in microbial communities were reflected in the plant’s internal physiology, as distinct hormonal and nutrient profiles were observed among cultivars with contrasting tillering capacities (Figs. 3, Tables S4–S5).

### Endogenous hormone profiles across varieties and tissues

Quantitative analysis of endogenous hormone levels revealed distinct hormonal patterns associated with sugarcane tillering capacity and tissue type (Fig. 3a, Table S4). High-tillering varieties (GT07168 and GL05136) exhibited elevated concentrations of growth-promoting hormones, particularly indole-3-acetic acid (IAA) and active cytokinins. GL05136 recorded the highest IAA content (9.55 ng/g FW) and trans-zeatin (tZ) level (0.57 ng/g FW), while GT07168 also maintained relatively high levels of these hormones. In contrast, low-tillering varieties (GT60 and ZZ6) displayed lower IAA concentrations and reduced active cytokinin levels despite some accumulation of cytokinin-glucosides (e.g., cZROG) in ZZ6, suggesting possible inactivation of cytokinin signaling. Additionally, abscisic acid (ABA) concentrations were significantly higher in low-tillering varieties, with GT60 showing the highest ABA content (6.84 ng/g FW) compared to a much lower level in GT07168 (3.57 ng/g FW). Gibberellin-related differences were also noted, with GA19 levels being elevated in GT60 but lower in high-tillering genotypes, indicating distinct hormonal balances that may influence tillering outcomes.

Further analysis comparing the main stem and tiller buds revealed tissue-specific hormonal distributions. In high-tillering varieties, tiller buds consistently exhibited higher IAA and tZ levels than the main stems. For instance, in GL05136, IAA increased from 8.97 ng/g FW in the main stem to 10.14 ng/g FW in the tiller buds, and a similar pattern was observed in GT07168. Active cytokinins, particularly tZ and cZR, were also relatively enriched in tiller tissues, supporting enhanced axillary bud activation. Conversely, ABA concentrations were markedly higher in tiller buds than in main stems, particularly in GL05136, where ABA content rose from 3.14 ng/g FW to 9.42 ng/g FW. Gibberellin precursor GA19 tended to be higher in main stems than in tiller buds, especially in GL05136. In the low-tillering variety GT60, hormonal differences between the main stem and tiller buds were less pronounced, and the cytokinin and auxin signaling appeared relatively weaker.

Together, these results suggest that high-tillering sugarcane varieties maintain a favorable hormonal environment for axillary bud outgrowth, characterized by higher levels of auxins and active cytokinins and reduced ABA accumulation, with these differences being particularly evident in the tiller buds compared to the main stems. The hormonal profiles suggest a physiological mechanism linking rhizosphere-derived signals to nutrient uptake and bud activation, implying that microbial-driven nutrient dynamics could shape hormonal equilibria controlling tiller outgrowth.

### Nutrient element profiles across varieties and tissues

Analysis of mineral element concentrations revealed distinct nutrient profiles associated with sugarcane tillering capacity (Fig. 3b, Table S5). High-tillering varieties (GT07168 and GL05136) generally exhibited higher nitrogen (N) and phosphorus (P) levels compared to low-tillering varieties (GT60 and ZZ6). Specifically, GL05136 showed the highest nitrogen content (0.76%) and relatively high phosphorus content (0.72%), while GT07168 displayed the highest phosphorus accumulation (0.89%). In contrast, GT60 and ZZ6 maintained lower N (0.46% and 0.44%, respectively) and P (0.60% and 0.68%, respectively) levels, indicating potentially limited nutrient availability for promoting axillary bud outgrowth.

Potassium (K) levels showed a more variable pattern. GT07168 recorded the highest K content (12.95%), followed by GT60 (11.59%) and ZZ6 (11.36%), while GL05136 exhibited the lowest K concentration (10.01%). Despite lower K, GL05136 maintained superior N and P nutrition, suggesting that balanced macronutrient acquisition rather than high potassium alone may favor tillering.

Regarding secondary nutrients, magnesium (Mg) and calcium (Ca) levels varied modestly among varieties. GL05136 exhibited relatively lower Mg (0.69%) but higher Ca (1.36%) concentrations. Conversely, GT60 and ZZ6 showed higher Mg levels but similar or slightly elevated Ca concentrations, suggesting different mineral homeostasis strategies.

Micronutrient analysis revealed that low-tillering varieties tended to accumulate more zinc (Zn) and manganese (Mn). ZZ6 had the highest Zn (16.48 mg/kg) and Mn (39.76 mg/kg) contents, whereas GL05136 and GT07168 maintained lower levels of these elements. High Zn and Mn accumulation may reflect stress responses rather than direct promotion of tillering.

These findings suggest that high-tillering varieties maintain superior nitrogen and phosphorus nutrition, potentially providing a more favorable environment for bud activation and shoot proliferation. In contrast, low-tillering varieties exhibit higher micronutrient accumulation, possibly linked to stress adaptation rather than enhanced growth.

### Microbial network architecture and tissue-specific environmental correlations

**Fig. 4:**
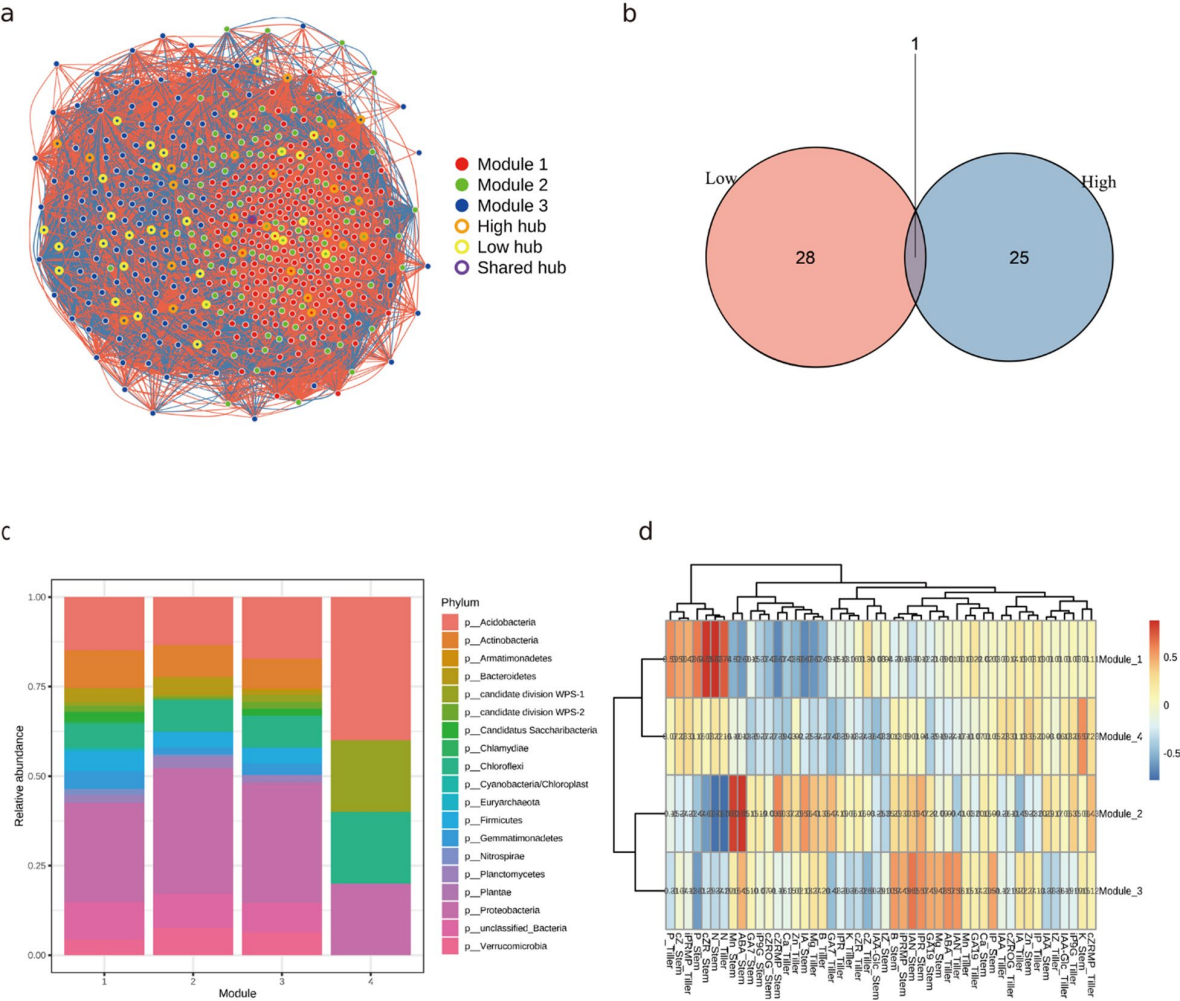
Microbial co-occurrence network architecture and environmental correlations in sugarcane rhizospheres. (**a**) The network graph shows distinct hub OTUs in high- and low-tillering cultivars. (**b**) Hub node distribution highlights genotype-specific microbial core structures. (**c**) Taxonomic composition of key network modules reflects functional compartmentalization. (**d**) Module–trait correlation heatmap reveals associations between network modules and plant nutrient/hormone profiles in different tissues, illustrating how microbial community structure is linked to sugarcane tillering physiology.

To further elucidate the interdependence among these factors, we integrated the microbial, nutrient, and hormone datasets. Network-level analyses revealed that specific microbial modules were strongly correlated with distinct nutrient and hormone patterns, indicating a systems-level coordination between the rhizosphere and plant physiological traits. Co-occurrence network analysis based on Spearman correlations (Fig. 4a) revealed distinct microbial interaction strategies between high- and low-tillering sugarcane cultivars. A total of 28 hub OTUs were exclusive to the low-tillering group, 25 were unique to the high-tillering group, and only one hub was shared between them (Fig. 4b), suggesting that divergent microbial core structures underpin the rhizosphere communities of different genotypes. In high-tillering varieties, hub nodes were more broadly distributed across multiple modules and were dominated by positive correlations, forming intermodular linkages indicative of cooperative microbial associations. In contrast, low-tillering networks were characterized by more localized hub clustering and a higher proportion of negative correlations, reflecting competitive or antagonistic microbial interactions that may restrict functional redundancy and network integration.

At the phylum level, each module exhibited distinct taxonomic signatures (Fig. 4c), suggesting functional compartmentalization. Module 1 was enriched in taxa typically associated with saprotrophic and nutrient-responsive groups, suggesting potential involvement in nitrogen-related processes.Module 2 consisted mainly of taxa commonly linked to stress-adapted microbial assemblages, indicating possible roles in organic matter turnover under stress-prone conditions. Module 3 was composed primarily of Chloroflexi and Acidobacteria, taxa associated with oligotrophic lifestyles and recalcitrant carbon degradation, potentially functioning in resource-limited or senescent root environments. Module 4 presented a mixed profile, including Proteobactceria, Firmicutes, and Gemmatimonadetes, suggesting a transitional module that bridges nutrient-rich and stress-prone soil zones. All functional interpretations above are putative and based on amplicon-inferred patterns; therefore, they require validation using higher-resolution metagenomic or metatranscriptomic approaches.

Correlation analysis between module eigengenes and environmental variables (Fig. 4d) revealed a high degree of coordination between rhizosphere microbial consortia and tissue-specific physiological signatures. Specifically, Module 1 emerged as a critical regulatory hub linked to active tiller development, showing strong positive correlations with nitrogen (N), phosphorus (P), and potassium (K) concentrations in the stem and with indole-3-acetic acid (IAA) levels in tiller buds, this suggests that the enrichment of Module 1 reflects a functional microbiome tailored to support the nutrient-hormonal balance required for axillary bud outgrowth. Module 2 served as a stress-associated signature characterizing low-tillering cultivars, being positively associated with calcium (Ca) and manganese (Mn) accumulation in tiller buds and with abscisic acid (ABA) levels in stems. These findings illustrate that the microbial environment in low-tillering genotypes is synchronized with growth-inhibitory signals. While these module–trait relationships are correlative, the consistent alignment across multiple physiological layers underscores a systems-level coupling between rhizosphere communities and sugarcane tillering performance. Module 3 showed negative correlations with boron (B), zinc (Zn) in the stem, and GA₁₉ in buds, a pattern consistent with low-nutrient or hormone-responsive physiological states. Module 4 correlated positively with iron (Fe) and magnesium (Mg) in stems and with the bioactive gibberellin GA₇ in buds, showing associations with redox-related and GA-associated traits that may correspond to microbial assemblages present in elongating stem tissues. These module–trait relationships are correlative rather than causal, and their functional interpretations remain provisional due to the inherent limitations of amplicon-based inference.

These results collectively highlight that rhizosphere microbial networks are structurally distinct between genotypes and tightly coupled with the spatial distribution of plant nutrients and hormones. Specifically, the identification of Module 1 as a positive regulator linked to high N, P, and K accumulation in the stem and elevated IAA levels in tiller buds provides a direct link between microbial hubs and the hormonal signaling required for axillary bud outgrowth. In contrast, the association between Module 2 and ABA accumulation suggests a microbial-mediated reinforcement of bud dormancy in low-tillering cultivars. Integrating network topology, taxonomic identity, and environmental responsiveness underscores a systems-level mechanism by which microbial communities may influence sugarcane tillering performance.

## Discussion

Collectively, these physiological differences in hormone and nutrient profiles suggested that tillering capacity might be modulated not only by intrinsic metabolic regulation but also by interactions with rhizosphere-associated microbes. By integrating microbiome, hormone, and nutrient datasets, our findings reveal that tillering capacity in sugarcane is not determined by a single factor but by multi-layered feedback among rhizosphere microbial networks, nutrient status, and hormonal signaling.

### Rhizosphere microbial community shifts in high- vs. low-tillering varieties

High-tillering cultivars maintained a more diverse and conserved rhizosphere microbial community structure, whereas low-tillering varieties exhibited greater variability and inconsistency among replicates. In ordination analyses (PCA), high-tillering genotypes clustered tightly, indicating similar community compositions, while low-tillering genotypes were more dispersed, reflecting greater microbiome heterogeneity. Additionally, high-tillering cultivars harbored a significantly larger number of unique operational taxonomic units (OTUs)—up to 562—compared to as few as 172 unique OTUs in low-tillering varieties. This enrichment suggests that high-tillering rhizospheres maintain a broader functional repertoire that could enhance nutrient acquisition and growth support, consistent with reports that greater microbial diversity improves soil functionality and plant performance^33^.

In contrast, low-tillering cultivars, with fewer unique OTUs, may lack certain beneficial microbial functions, relying on a narrower and potentially less supportive microbial community. These findings align with previous studies showing that plant genotype and vigor strongly influence rhizosphere microbiome structure^34^. For instance, stress-tolerant or high-yielding sugarcane cultivars have been reported to sustain more stable and beneficial microbial communities under adverse conditions, whereas sensitive cultivars often experience community shifts toward less favorable profiles^24^. Thus, high-tillering varieties in our study likely possess an enhanced ability to recruit and maintain growth-promoting microorganisms, contributing to their superior tillering capacity.

Importantly, a conserved core microbiome comprising 518 OTUs was shared across all varieties, indicating that specific bacterial taxa are ubiquitously associated with sugarcane roots regardless of tillering performance. However, high-tillering cultivars exhibited distinct expansions of specific microbial groups beyond this core. Such variety-specific assemblages likely arise from differences in root exudate composition and physiology. Plants are known to modulate rhizosphere microbiomes through selective exudation, influencing the recruitment of beneficial microbes^35^. High-tillering sugarcane varieties may secrete exudates that preferentially attract plant growth-promoting rhizobacteria (PGPR), enriching taxa involved in nutrient cycling and hormonal stimulation, while low-tillering varieties may fail to promote such beneficial associations to the same extent.

This interpretation is supported by prior findings in wheat, where rhizobacteria associated with high-yielding varieties exhibit enhanced traits such as auxin production, nitrogen fixation, and phosphate solubilization^36^. Similarly, the enriched microbial communities observed in high-tillering sugarcane cultivars likely contribute directly to their improved growth and tillering performance. In summary, the observed differences in rhizosphere microbiome composition and diversity suggest that microbiota are integral components of tillering capacity, with high-tillering varieties benefiting from a more diverse and functionally tailored microbial consortium.

### Functional roles of key rhizosphere taxa in growth promotion

Taxonomic profiling revealed that Proteobacteria, Acidobacteriota, and Chloroflexi were the dominant phyla differentiating the rhizospheres of high- and low-tillering sugarcane varieties. While Proteobacteria were abundant across all samples, their dominance was significantly higher in low-tillering cultivars. In contrast, high-tillering rhizospheres were enriched in Acidobacteriota, Chloroflexi, and Planctomycetota, suggesting a microbiome more geared toward nutrient cycling and soil stability. These patterns imply distinct ecological strategies, with low-tillering plants favoring stress-adapted communities and high-tillering plants selecting for growth-promoting microbes.

Acidobacteriota were notably more abundant in high-tillering varieties. Once considered mainly oligotrophic, certain Acidobacteria are now recognized as plant growth-promoting rhizobacteria (PGPR), capable of enhancing biomass^37^, degrading complex organic materials^38^, and cycling nitrogen^39^ and phosphorus^40^. The higher prevalence of *Acidobacterium* in high-tillering rhizospheres supports the idea that these communities actively enhance nutrient turnover and auxin production, fostering tiller development.

Similarly, Chloroflexi were enriched in high-tillering varieties, with known roles in decomposing recalcitrant compounds^41^ and cycling carbon^42^ and nitrogen^43^. Their ability to thrive in stable soil aggregates^44^ suggests they contribute to long-term soil fertility and resource mobilization, supporting sustained growth.

In contrast, Proteobacteria were disproportionately dominant in low-tillering rhizospheres. Although some Proteobacteria act as PGPR^45^, their overrepresentation—especially of stress-tolerant groups like *Rhodanobacter*^46^ and unclassified Rhodospirillales^47^—may indicate a microbial community adapted to stress rather than growth promotion^48^. These low-tillering rhizospheres prioritized defense-oriented functions, such as antibiotic production and heavy metal detoxification.

Notably, despite a lower overall abundance of Proteobacteria, high-tillering plants selectively enriched beneficial genera such as *Bradyrhizobium*^49^ and *Streptomyces*^50^, known for nitrogen fixation, phytohormone production, and pathogen suppression.

Together, these findings highlight that microbial community composition—not merely diversity—plays a crucial role. High-tillering cultivars foster functional microbiomes that enhance nutrient supply and signaling for tiller initiation, whereas low-tillering cultivars harbor communities skewed toward survival under stress, potentially limiting growth potential.

### Microbial functional pathways correlated with tillering performance

Predictive functional profiling revealed that rhizosphere microbiomes associated with high-tillering sugarcane varieties are functionally geared toward growth promotion, whereas those of low-tillering varieties are oriented toward stress adaptation. High-tillering microbiomes were enriched in pathways related to nitrogen fixation, phosphate solubilization, carbohydrate degradation, and auxin biosynthesis—functions intimately linked to nutrient acquisition^51^ and tiller development^52^. The presence of nitrogen-cycling pathways, consistent with higher plant tissue N content, suggests that microbial nitrogen fixation may enhance tiller formation. Similarly, enrichment in phosphate solubilization pathways likely facilitates greater phosphorus availability, supporting early shoot development, while elevated carbohydrate metabolism functions indicate active organic matter decomposition, promoting sustained nutrient release. The increased microbial auxin biosynthesis prediction further suggests that rhizosphere-derived hormonal cues may stimulate axillary bud activation and outgrowth.

In contrast, low-tillering cultivars’ microbiomes were enriched in pathways associated with oxidative stress response, heavy metal detoxification, and antibiotic resistance. While beneficial for microbial survival, these functions are less directly supportive of plant growth. The dominance of stress-response pathways implies that microbial communities around low-tillering roots are adapted to cope with reactive oxygen species, metal toxicity, and microbial competition, potentially reflecting harsher microenvironments. Such defensive functional skewing likely limits the microbial contribution to nutrient cycling and growth stimulation, constraining tillering capacity.

These functional predictions align with observed phenotypic outcomes: high-tillering sugarcane cultivars benefit from microbiomes that enhance nutrient availability and provide growth-promoting signals, whereas low-tillering cultivars harbor microbiomes primarily focused on stress endurance.

### Endogenous hormone profiles in High- vs. Low-tillering varieties

Endogenous hormone profiling revealed that high- and low-tillering sugarcane cultivars exhibit distinct hormonal balances, likely contributing to their contrasting tillering capacities. High-tillering varieties (GT07168 and GL05136) displayed elevated levels of growth-promoting hormones, particularly auxin (IAA) and cytokinins, within tiller buds. In contrast, low-tillering varieties (GT60 and ZZ6) accumulated higher growth-inhibitory hormone abscisic acid (ABA) levels and showed reduced active cytokinin and auxin concentrations.

In high-tillering cultivars, tiller buds exhibited higher IAA content than stems, suggesting active auxin production supporting bud outgrowth rather than classical apical dominance suppression^53^. Concurrently, elevated trans-zeatin (tZ) levels in buds and stems of high-tillering varieties reinforced a hormonal environment favoring axillary bud activation. By contrast, low-tillering cultivars accumulated inactive cytokinin conjugates (e.g., cZROG), further weakening bud activation signals. Additionally, ABA levels were significantly higher in low-tillering genotypes, consistent with ABA’s known role in promoting bud dormancy and stress signaling^54^. In GT60, for example, ABA levels remained high across both stems and buds, potentially maintaining a systemic suppression of tiller growth, whereas, in high-tillering cultivars, ABA was generally lower and spatially regulated.

Gibberellin (GA) metabolism also differed: GT60 showed higher GA₁₉ accumulation, suggesting potential disruptions in GA activation pathways that may reinforce apical dominance. While GAs primarily promote stem elongation, excessive GA precursors without efficient conversion to active forms could contribute to inhibited lateral bud development^55^.

Overall, these hormonal disparities align with the observed phenotypic differences. High-tillering cultivars maintain a hormonal milieu characterized by enhanced auxin and cytokinin activity in buds and reduced systemic stress signaling, facilitating active tiller emergence. In contrast, low-tillering cultivars display hormonal profiles indicative of stronger apical dominance, elevated stress responses, and suppressed bud activation. These findings reinforce the critical role of the auxin–cytokinin–ABA balance in modulating sugarcane tillering and highlight endogenous hormone profiles as key determinants of branching potential, consistent with hormonal paradigms observed in other cereals such as rice and wheat^56^.

### Nutrient profiles associated with tillering capacity

Nutrient analysis revealed distinct nutritional patterns between High- and low-tillering sugarcane cultivars, underscoring the critical role of macronutrient status in supporting tillering. High-tillering varieties (GL05136 and GT07168) exhibited significantly higher tissue concentrations of nitrogen (N) and phosphorus (P) compared to low-tillering varieties (GT60 and ZZ6), suggesting that sufficient N and P availability is essential for vigorous tiller development. N content reached ~ 0.76% in GL05136, whereas low-tillering varieties had only ~ 0.44–0.46%. Given N’s role in amino acid synthesis and vegetative growth, these findings align with previous reports linking enhanced N nutrition to increased tillering in cereals^14^.

Similarly, P concentrations were higher in high-tillering plants (~ 0.89% vs. ~ 0.60% in low-tillering varieties). Phosphorus is crucial for energy transfer and meristem activity, and its sufficiency likely promoted robust basal shoot growth and tiller emergence^57^. The concurrent enrichment of N and P in high-tillering cultivars indicates a synergistic effect, providing metabolic energy and building blocks necessary for multiple tiller formation.

Potassium (K) did not show a consistent relationship with tillering; although GT07168 exhibited high K (~ 12.95%), GL05136 achieved high tillering with comparatively low K (~ 10.0%), suggesting that K was sufficient across genotypes and not a primary driver of tiller number under these conditions. Calcium (Ca) and magnesium (Mg) variations were moderate and not associated with tillering outcomes, implying that these nutrients were generally non-limiting.

Interestingly, low-tillering cultivars accumulated higher levels of zinc (Zn) and manganese (Mn), with ZZ6 showing the highest concentrations. While Zn and Mn are vital in trace amounts, their excessive accumulation may reflect stress conditions rather than improved growth. Possible explanations include a dilution effect (less biomass) or stress-induced micronutrient uptake, consistent with the stress-oriented rhizosphere functional profiles observed in low-tillering genotypes.

Overall, superior tillering capacity in sugarcane is associated with high N and P availability and balanced micronutrient uptake rather than sheer accumulation of nutrients. Ensuring sufficient N and P supply appears critical for maximizing tiller numbers, while excessive micronutrient accumulation without macronutrient sufficiency may correspond to suppressed tillering performance.

### Network-structured microbial–environmental interactions shape sugarcane tillering potential

The contrasting co-occurrence network structures between high- and low-tillering sugarcane cultivars reflect fundamentally different rhizosphere assembly strategies^58^. High-tillering varieties exhibit a modular, cooperative microbial architecture with diverse hub taxa—25 hub OTUs unique to high-tillering plants and more evenly distributed across modules—while low-tillering cultivars develop stress-oriented networks characterized by hub aggregation, reduced modular complexity, and a higher prevalence of negative correlations. These structural differences suggest that microbial connectivity and redundancy may be critical for sustaining rhizosphere functions relevant to tiller formation and buffering against environmental perturbations^59^.

Module-level taxonomic partitioning revealed clear differences among cultivars and suggested that microbial assemblages may occupy distinct ecological niches within the rhizosphere. Modules enriched in taxa that are commonly associated with nutrient turnover or phytohormone-related processes in previous studies, such as Proteobacteria and Bacteroidota (Module 1), were more abundant in high-tillering cultivars. These modules showed correlations with elevated levels of macronutrients (N, P, K) and growth-promoting hormones such as IAA and GA₇, especially in tiller buds and stem tissues^60,61^. For instance, GL05136, a high-tillering genotype, displayed significant enrichment of Module 1 and corresponding increases in IAA and nutrient accumulation in bud tissue, indicating a nutrient–hormone pattern consistent with active bud growth. While this pattern suggests possible microbially aligned nutrient availability, these relationships remain correlative within the context of the present dataset. Module 4, which contained multiple bacterial lineages also documented in nutrient-associated or stress-responsive environments, showed positive correlations with Fe, Mg, and GA₇^60,61^. These correlations may reflect microbial assemblages that occur alongside nutrient and hormone states characteristic of elongating stem tissues. Overall, these module–trait associations should be interpreted as correlations rather than direct functional interactions, given the inherent limitations of amplicon-based inference.

In contrast, low-tillering cultivars (e.g., GT60 and ZZ6) exhibited enrichment of stress-associated modules, particularly Module 2 (Actinobacteria and Firmicutes-dominated) and Module 3 (Acidobacteriota and Chloroflexi). These modules correlated with increased ABA concentrations and higher Mn and Zn accumulation in tiller buds or stems^62^. ABA, known for its inhibitory role in bud outgrowth and its involvement in stress signaling, was substantially elevated in these genotypes^63^, suggesting that the microbial environment may reinforce physiological constraints that suppress tillering. The presence of micronutrient imbalances further indicates potential oxidative stress or metal-associated antagonism that may alter bud fate^60,61^.

These findings support a systems-level model in which sugarcane tillering capacity emerges from tightly coupled interactions between genotype-specific microbial networks and spatially distinct physiological signals^64^. As evidenced by the strong correlations in Fig. 4d, rhizosphere microbial modularity is functionally aligned with host tissue nutrient status and hormone distribution, where the cooperative Module 1 specifically facilitates tillering by synchronizing with elevated N, P, and IAA levels. Rhizosphere microbial modularity is taxonomically structured and functionally aligned with host tissue nutrient status and hormone distribution. High-tillering genotypes appear to benefit from feedback loops between cooperative microbial networks and favorable physiological states, whereas low-tillering cultivars may be constrained by feedbacks that emphasize defense or metabolic conservation. Strategically modifying rhizosphere communities to enhance nutrient availability and rebalance hormone dynamics could represent a promising avenue to promote tillering in sugarcane.

Although functional annotations were derived from amplicon-based inference, the observed associations were supported by independent physiological data, including nutrient accumulation patterns and endogenous hormone profiles, strengthening the biological relevance of our findings.

## Conclusion

Rhizosphere microbial networks, nutrient status, and hormone signaling jointly regulate sugarcane tillering. High-tillering cultivars support cooperative microbial consortia aligned with growth-promoting signals while low-tillering cultivars harbor stress-oriented communities linked to ABA and micronutrient accumulation. Microbial network topology and module composition reflect and reinforce plant physiological states. These findings highlight the potential of managing rhizosphere microbiota to enhance tillering through integrated nutrient–hormone–microbe interactions.

## Supplementary Information

Below is the link to the electronic supplementary material.

Supplementary Material 1

## Data Availability

The datasets generated and/or analysed during the current study are available in the National Microbiology Data Center (NMDC) repository, with accession number [NMDC10020016] (https://nmdc.cn/resource/genomics/project/detail/NMDC10020016).
