# Supplementary material for: Rhizosphere microbiome dynamics and hormonal interactions regulating tiller development in sugarcane cultivars
Source: Sci Rep. 2026 Mar 22;16:14500. doi: 10.1038/s41598-026-38474-y (PMC13149797; doi:10.1038/s41598-026-38474-y)
Supplement: Supplementary file 1 — Supplementary Material 1 [file 41598_2026_38474_MOESM1_ESM.docx]

Table S1 Mobile phase composition and gradient conditions for LC-MS/MS analysis.

| Time (min) | Flow (ml/min) | 0.1%formic acid solution (%) | Methanol (%) |
| --- | --- | --- | --- |
| 0 | 0.3 | 90 | 10 |
| 3 | 0.3 | 5 | 95 |
| 4 | 0.3 | 90 | 10 |
| 5 | 0.3 | 90 | 10 |

Table S2 Precursor/product ion transitions and collision energies for targeted compounds in LC-MS/MS analysis.

| Analytes | Scan mode | Precursor ions (*m/z*) | Product ions (*m/z*, **quantitative ion**/qualitative ion) | Collision energy (V) |
| --- | --- | --- | --- | --- |
| Salvianolic Acid B | Negative | 717.133 | **320.967**, 518.967 | 31.69, 16.42 |
| Cinnamic Acid | Positive | 148.967 | **84.8**, 116.883 | 11.72, 5.3 |
| 1-Aminocyclopropanecarboxylic Acid | Positive | 101.883 | **56.217**, 73.883 | 13.54, 13.09 |
| Quinic acid | Negative | 190.967 | **84.883**, 92.883 | 20.92, 21.43 |
| trans-Zeatin | Positive | 220.05 | **135.967**, 202.05 | 17.43, 12.38 |
| Chlorogenic Acid | Negative | 353.05 | **135.05**, 178.883 | 33, 12.94 |
| Protamine Sulfate | Negative | 136.883 | **91.883**, 107.883 | 23.5, 22.99 |
| Caffeic Acid | Negative | 178.883 | **90.883**, 134.883 | 16.98, 11.98 |
| Gibberellin | Negative | 345.05 | **221.05**, 239.05 | 23.75, 13.54 |
| Sinapic acid | Positive | 225.05 | **206.967**, 208.967 | 9.75, 15.46 |
| Phenolic Acid | Negative | 162.967 | **92.883**, 118.967 | 31.23, 13.74 |
| Rosmarinic Acid | Negative | 358.967 | **160.883**, 196.883 | 17.89, 15.56 |
| Rutin | Positive | 611.217 | **302.967**, 465.05 | 20.21, 11.42 |
| Salvianolic Acid A | Negative | 493.05 | **184.883**, 294.967 | 25.83, 15.36 |
| 3-Indoleacetic Acid | Positive | 175.967 | **102.967**, 129.967 | 31.74, 14.86 |
| Astragalin | Negative | 447.05 | **254.883**, 283.883 | 38.31, 26.03 |
| Abscisic Acid | Negative | 263.133 | **152.967**, 219.05 | 9.75, 11.57 |
| Coumalic Acid | Positive | 141.05 | **54.883**, 86.967 | 12.18, 9.25 |
| Quercetin | Negative | 300.967 | **150.883**, 273.05 | 20.82, 16.12 |
| Salicylic Acid | Negative | 136.883 | **64.883**, 92.883 | 28.61, 15.82 |
| Strigolactone analog | Positive | 299.05 | **96.883**, 281.05 | 17.54, 6.21 |
| Dihydrojasmonic Acid | Negative | 211.05 | **58.883**, 110.967 | 13.14, 21.07 |
| Brassinolide | Positive | 481.383 | **349.217**, 445.383 | 13.64, 11.02 |
| Methyl jasmonate | Positive | 225.05 | **147.05**, 150.967 | 13.14, 11.77 |

Table S3 Relative abundance (%) of major bacterial phyla in the rhizosphere of high- and low-tillering sugarcane cultivars.

|  | Proteobacteria (%) | Acidobacteriota (%) | Chloroflexi (%) | Actinobacteriota (%) | Verrucomicrobiota (%) | Firmicutes (%) | Bacteroidota (%) | Planctomycetota (%) | Myxococcota (%) | Gemmatimonadota (%) | Patescibacteria (%) | WPS-2 (%) | Bdellovibrionota (%) | unclassified_Bacteria (%) | Other (%) |
| --- | --- | --- | --- | --- | --- | --- | --- | --- | --- | --- | --- | --- | --- | --- | --- |
| GT60 | 46.71±2.96 | 8.13±0.22 | 15.06±2.5 | 4.1±0.43 | 4.58±0.73 | 3.04±0.44 | 4.58±0.62 | 3.94±0.67 | 2.78±0.19 | 1.63±0.23 | 2.59±0.43 | 0.22±0.04 | 0.24±0.01 | 0.45±0.06 | 1.95±0.13 |
| ZZ6 | 63.15±1.21 | 9.66±0.5 | 7.31±1.08 | 4.46±0.64 | 1.01±0.06 | 2.73±0.52 | 3.76±0.24 | 1.36±0.07 | 0.82±0.07 | 1.55±0.36 | 1.75±0.17 | 0.77±0.34 | 0.75±0.47 | 0.52±0.04 | 0.4±0.04 |
| GT07168 | 24.21±2.59 | 30.04±0.93 | 20.37±0.7 | 4.66±0.25 | 3.84±0.25 | 2.66±0.12 | 1.25±0.07 | 3.53±0.28 | 1.81±0.19 | 1.77±0.13 | 0.86±0.08 | 1.88±0.14 | 0.16±0.02 | 0.81±0.02 | 2.16±0.21 |
| GL05136 | 25.14±1.98 | 29.36±3.79 | 13.13±0.66 | 5.9±1.05 | 4.33±0.22 | 4.43±0.64 | 3.15±1.46 | 3.13±0.29 | 2.03±0.05 | 2.37±0.49 | 1±0.07 | 2.23±0.08 | 0.29±0.02 | 0.97±0.1 | 2.55±0.08 |

Table S4 Endogenous hormone (ng/g FW) profiles in different sugarcane cultivars and tissues.

|  | IAA (ng/g FW) | IAA-Glc (ng/g FW) | IAN (ng/g FW) | IA (ng/g FW) | IP (ng/g FW) | iP9G (ng/g FW) | IPR (ng/g FW) | iPRMP (ng/g FW) | cZ (ng/g FW) | cZR (ng/g FW) | cZRMP (ng/g FW) | cZROG (ng/g FW) | tZ (ng/g FW) | ABA (ng/g FW) | GA19 (ng/g FW) | GA7 (ng/g FW) |
| --- | --- | --- | --- | --- | --- | --- | --- | --- | --- | --- | --- | --- | --- | --- | --- | --- |
| GT60_stem | 7.42±1.2a | 20.96±9.4a | 0.5±0.08a | 1.3±0.68a | 0.19±0.01a | 0.2±0.09b | 0.03±0.01a | 0.09±0.04a | 0.05±0.01b | 0.23±0.02ab | 0.14±0.14a | 2.58±0.57a | 0.41±0.12ab | 6.62±0.58ab | 2.13±0.32a | 0.18±0.05ab |
| GT60_tiller | 8.68±1.14a | 14.11±6.13a | 0.46±0.11a | 0.46±0.46a | 0.11±0.05a | 0.04±0.04b | 0.01±0.01a | 0±0a | 0.05±0.03ab | 0.23±0.01ab | 0.12±0.12a | 2.04±0.84a | 0.31±0.14b | 7.06±0.16ab | 0.85±0.51ab | 0.11±0.09ab |
| ZZ6_stem | 8.35±0.15a | 13.26±13.26a | 0.37±0.19a | 1.29±0.73a | 0.12±0.04a | 0.13±0.08ab | 0.03±0.01a | 0.06±0.03a | 0.07±0.02ab | 0.17±0b | 0.34±0.17a | 4.14±1.86a | 0.4±0.08ab | 8.44±2.36ab | 1.04±0.52ab | 0.21±0.01ab |
| ZZ6_tiller | 8.16±1.06a | 28.56±14.28a | 0.15±0.07a | 0±0a | 0.09±0.02a | 0.09±0.05ab | 0.05±0.03a | 0±0a | 0.09±0.01ab | 0.34±0.08ab | 0.39±0.22a | 3.66±1.33a | 0.59±0.18ab | 4.49±0.17ab | 0.21±0.21b | 0.3±0.09ab |
| GT07168_stem | 8±0.98a | 17.53±6.49a | 0.28±0.15a | 0.61±0.42a | 0.14±0.03a | 0.25±0.07ab | 0.02±0.01a | 0.03±0.03a | 0.08±0.04ab | 0.34±0.03ab | 0±0a | 1.93±0.58a | 0.42±0.07ab | 3.75±0.48ab | 0.41±0.41b | 0.07±0.04b |
| GT07168_tiller | 10.24±1.85a | 19.74±3.86a | 0.24±0.01a | 0.54±0.31a | 0.43±0.29a | 0.4±0.21a | 0.03±0.02a | 0.09±0.09a | 0.1±0.03ab | 0.36±0.07a | 0.16±0.16a | 2.36±0.22a | 0.47±0.05ab | 3.4±0.72ab | 0.56±0.56b | 0.09±0.01b |
| GL05136_stem | 8.97±2.69a | 20.13±9.32a | 0.25±0.13a | 0±0a | 0.13±0.06a | 0±0b | 0.07±0.07a | 0.1±0.1a | 0.13±0.01a | 0.39±0.07a | 0±0a | 2.83±0.27a | 0.7±0.05a | 3.14±0.24b | 1.4±0.36ab | 0.36±0.14a |
| GL05136_tiller | 10.14±0.57a | 20.77±10.45a | 0.37±0.02a | 0.44±0.44a | 0.08±0a | 0±0b | 0.02±0.02a | 0±0a | 0.12±0.02ab | 0.25±0.09ab | 0.13±0.13a | 3.57±0.85a | 0.44±0.06ab | 9.42±4.45a | 0.62±0.36b | 0.23±0.1ab |

Note: Values are presented as the mean ± standard error (SE). Lowercase letters indicate statistically significant differences among groups according to one-way ANOVA followed by Duncan’s multiple-range test (p < 0.05); “a” represents the highest mean value, and subsequent letters indicate decreasing means.

Table S5 Nutrient element concentrations (%) in sugarcane cultivars with contrasting tillering capacities.

|  | N (%) | P (%) | K (%) | Mg (%) | Ca (%) | B (mg/kg) | Zn (mg/kg) | Mn (mg/kg) |
| --- | --- | --- | --- | --- | --- | --- | --- | --- |
| GT60_stem | 0.51±0.01d | 0.61±0.05b | 13.18±2.46a | 0.86±0.07a | 1.37±0.12ab | 17.06±1.5a | 14.85±2.13ab | 36.33±7.98ab |
| GT60_tiller | 0.41±0.01e | 0.59±0.05b | 9.99±2.1a | 0.8±0.1a | 1.38±0.08ab | 14.37±0.83a | 13.87±0.31ab | 36.22±4.4ab |
| ZZ6_stem | 0.48±0.01d | 0.73±0.02ab | 10.9±3.27a | 0.7±0.11a | 1.49±0.19ab | 15.38±0.8a | 15.43±1.66ab | 40.94±4.29ab |
| ZZ6_tiller | 0.4±0.01e | 0.62±0.06b | 11.83±3.52a | 0.87±0.01a | 1.74±0.03a | 15.76±1.59a | 17.53±1.09a | 38.59±6.31ab |
| GT07168_stem | 0.63±0.01c | 0.96±0.12a | 13.75±3.21a | 0.74±0.06a | 1.18±0.08b | 14.46±3.29a | 14.3±1.4ab | 30.2±2.62ab |
| GT07168_tiller | 0.69±0.02b | 0.81±0.18ab | 12.15±0.95a | 0.69±0.08a | 1.28±0.24b | 12.67±2.07a | 14.27±2.66ab | 29.88±7.27ab |
| GL05136_stem | 0.63±0.02c | 0.82±0.02ab | 11.81±1.6a | 0.7±0.04a | 1.41±0.03ab | 13.36±0.95a | 14.98±0.34ab | 23.91±2.02b |
| GL05136_tiller | 0.89±0.02a | 0.63±0.03b | 8.21±0.35a | 0.69±0.01a | 1.31±0.04b | 12.37±0.38a | 11.87±1.43b | 48.19±8.83a |

Note: Values are presented as the mean ± standard error (SE). Lowercase letters indicate statistically significant differences among groups according to one-way ANOVA followed by Duncan’s multiple-range test (p < 0.05); “a” represents the highest mean value, and subsequent letters indicate decreasing means.
